# 

**Published:** 1855-11

**Authors:** 


VOL. XXXII.] [NO. 191.

THE

### WESTERN JOURNAL

OF

### MEDICINE AND SURGERY.

NEW SERIES. NOVEMBER, 1855. VOL. IV.—NO. 11.

EDITED BY
LUNSFORD P. YANDELL, M. D.
PROFESSOR OF PHYSIOLOGY AND PATHOLOGICAL ANATOMY, IN THE UNIVERSITY OF LOUISVILLE.

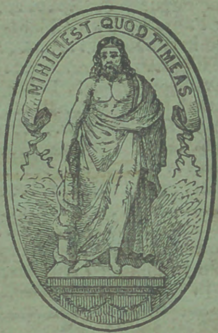

LOUISVILLE, KY:
PRINTED AND PUBLISHED BY J. F. BRENNAN,
*Cor. of Market & First Streets.*

☞PRICE, $3 A YEAR—INVARIABLY IN ADVANCE.—POSTAGE, 2 CENTS A NUMBER.☜

